# Supplementary material for: Modeling glioblastoma heterogeneity as a dynamic network of cell states
Source: Mol Syst Biol. 2021 Sep 16;17(9):e10105. doi: 10.15252/msb.202010105 (PMC8444284; doi:10.15252/msb.202010105)
Supplement: Supplementary file 6 — Source Data for Figure 5 [file MSB-17-e10105-s004.zip › Figure5A_sourcedata/GSEA_3071/nosigdb_stateI.GseaPreranked.1622795145664/ANASTASSIOU_MULTICANCER_INVASIVENESS_PMID_22208948.html]

Details for gene set ANASTASSIOU\_MULTICANCER\_INVASIVENESS\_PMID\_22208948[GSEA]

|  || Dataset | state13071 |
| Phenotype | NoPhenotypeAvailable |
| Upregulated in class | na\_pos |
| GeneSet | ANASTASSIOU\_MULTICANCER\_INVASIVENESS\_PMID\_22208948 |
| Enrichment Score (ES) | 0.82403654 |
| Normalized Enrichment Score (NES) | 3.8327732 |
| Nominal p-value | 0.0 |
| FDR q-value | 0.0 |
| FWER p-Value | 0.0 |
Table: GSEA Results Summary

  

Fig 1: Enrichment plot: ANASTASSIOU\_MULTICANCER\_INVASIVENESS\_PMID\_22208948      
 Profile of the Running ES Score & Positions of GeneSet Members on the Rank Ordered List

  

| PROBE | GENE SYMBOL | GENE\_TITLE | RANK IN GENE LIST | RANK METRIC SCORE | RUNNING ES | CORE ENRICHMENT || 1 | COL3A1 |  |  | 3 | 2.646 | 0.0592 | Yes |
| 2 | LUM |  |  | 4 | 2.203 | 0.1092 | Yes |
| 3 | DCN |  |  | 5 | 2.010 | 0.1548 | Yes |
| 4 | COL1A1 |  |  | 6 | 2.008 | 0.2004 | Yes |
| 5 | PDGFRB |  |  | 9 | 1.893 | 0.2427 | Yes |
| 6 | FN1 |  |  | 11 | 1.769 | 0.2826 | Yes |
| 7 | COL6A3 |  |  | 13 | 1.717 | 0.3213 | Yes |
| 8 | COL5A1 |  |  | 14 | 1.706 | 0.3600 | Yes |
| 9 | PCOLCE |  |  | 15 | 1.696 | 0.3985 | Yes |
| 10 | BGN |  |  | 24 | 1.568 | 0.4317 | Yes |
| 11 | POSTN |  |  | 32 | 1.494 | 0.4636 | Yes |
| 12 | TNFAIP6 |  |  | 36 | 1.470 | 0.4961 | Yes |
| 13 | COL11A1 |  |  | 37 | 1.468 | 0.5294 | Yes |
| 14 | COL5A2 |  |  | 48 | 1.373 | 0.5577 | Yes |
| 15 | CDH11 |  |  | 59 | 1.296 | 0.5843 | Yes |
| 16 | COL6A2 |  |  | 67 | 1.237 | 0.6103 | Yes |
| 17 | SERPINF1 |  |  | 71 | 1.211 | 0.6369 | Yes |
| 18 | ASPN |  |  | 92 | 1.117 | 0.6565 | Yes |
| 19 | THY1 |  |  | 94 | 1.114 | 0.6815 | Yes |
| 20 | SPARC |  |  | 106 | 1.067 | 0.7025 | Yes |
| 21 | FBN1 |  |  | 138 | 0.944 | 0.7150 | Yes |
| 22 | INHBA |  |  | 140 | 0.941 | 0.7361 | Yes |
| 23 | SNAI2 |  |  | 142 | 0.940 | 0.7571 | Yes |
| 24 | GLT8D2 |  |  | 181 | 0.856 | 0.7656 | Yes |
| 25 | VCAN |  |  | 197 | 0.833 | 0.7802 | Yes |
| 26 | NUAK1 |  |  | 232 | 0.763 | 0.7877 | Yes |
| 27 | OLFML2B |  |  | 233 | 0.763 | 0.8051 | Yes |
| 28 | AEBP1 |  |  | 236 | 0.761 | 0.8217 | Yes |
| 29 | MMP2 |  |  | 284 | 0.697 | 0.8240 | Yes |
| 30 | NTM |  |  | 427 | 0.572 | 0.7961 | No |
| 31 | EDNRA |  |  | 479 | 0.529 | 0.7934 | No |
| 32 | RCN3 |  |  | 579 | 0.466 | 0.7755 | No |
| 33 | LOXL2 |  |  | 589 | 0.459 | 0.7833 | No |
| 34 | COPZ2 |  |  | 650 | 0.432 | 0.7759 | No |
| 35 | TIMP3 |  |  | 773 | 0.391 | 0.7496 | No |
| 36 | ADAM12 |  |  | 776 | 0.390 | 0.7579 | No |
| 37 | ITGBL1 |  |  | 784 | 0.389 | 0.7647 | No |
| 38 | MFAP5 |  |  | 2104 | -0.335 | 0.3925 | No |
| 39 | TMEM158 |  |  | 2900 | -0.543 | 0.1759 | No |
Table: GSEA details [plain text format]

  

Fig 2: ANASTASSIOU\_MULTICANCER\_INVASIVENESS\_PMID\_22208948: Random ES distribution      
 Gene set null distribution of ES for **ANASTASSIOU\_MULTICANCER\_INVASIVENESS\_PMID\_22208948**
